# Numb Isoforms Deregulation in Medulloblastoma and Role of p66 Isoform in Cancer and Neural Stem Cells

**DOI:** 10.3389/fped.2018.00315

**Published:** 2018-11-01

**Authors:** Luana Abballe, Angela Mastronuzzi, Evelina Miele, Andrea Carai, Zein Mersini Besharat, Marta Moretti, Enrico De Smaele, Felice Giangaspero, Franco Locatelli, Elisabetta Ferretti, Agnese Po

**Affiliations:** ^1^Department of Experimental Medicine, Sapienza University, Rome, Italy; ^2^Department of Pediatric Hematology/Oncology and Cellular and Gene Therapy, Bambino Gesù Children's Hospital, Rome, Italy; ^3^Neurosurgery Unit, Department of Neuroscience and Neurorehabilitation, Bambino Gesù Children's Hospital, Rome, Italy; ^4^Department of Molecular Medicine, Sapienza University, Rome, Italy; ^5^Department of Radiological, Oncological and Pathological Science, Sapienza University, Rome, Italy; ^6^IRCCS Neuromed, Pozzilli, Italy; ^7^Department of Paediatrics, University of Pavia, Pavia, Italy

**Keywords:** medulloblastoma, numb, NUMB isoforms, cancer stem cells, neural stem cells, sonic hedgehog signaling

## Abstract

Numb is an intracellular protein with multiple functions. The two prevalent isoforms, Numb p66 and Numb p72, are regulators of differentiation and proliferation in neuronal development. Additionally, Numb functions as cell fate determinant of stem cells and cancer stem cells and its abnormal expression has been described in several types of cancer. Involvement of deregulated Numb expression has been described in the malignant childhood brain tumor medulloblastoma, while Numb isoforms in these tumors and in cancer stem-like cells derived from them, have not been studied to date. Here we show that medulloblastoma stem-like cells and cerebellar neuronal stem cells (NSCs) express Numb p66 where its expression tampers stemness features. Furthermore, medulloblastoma samples evaluated in this study express decreased levels of Numb p66 while overexpressed Numb p72 compared with normal tissues. Our results uncover different roles for the two major Numb isoforms examined in medulloblastoma and a critical role for Numb p66 in regulating stem-like cells and NSCs maintenance.

## Introduction

Medulloblastoma (MB) arises in the cerebellum and is the most common malignant pediatric brain tumor (1, 2), originating from both granule cell progenitor (GCPs) and neural stem cells (3). Human medulloblastoma is divided in at least 4 molecular subgroups (WNT, SHH, Group 3, and Group 4) which are characterized by different patterns of gene expression, genetic aberrations, and clinical outcomes (4). SHH MBs account for roughly 27% of tumors and are characterized by aberrant activation of the Sonic hedgehog (Shh) pathway. This aberrant activation is mostly achieved by genetic loss of negative regulators (i.e., Ptch) or amplification of positive regulators (i.e., Gli2) (4–6).

Cancer subpopulations with stemness features (stem-like cells, SLCs) are considered the ultimate reservoir of cancer cells and have been isolated and characterized in several solid tumors, including brain tumors (7). In our previous studies we isolated and characterized cerebellar NSCs and SLCs of human and murine origin and we showed that Shh is a major driver of stemness in this context, through the transcriptional activity of the transcription factor Gli1 and the post-transcriptional regulation of Gli1 activity (8–12).

Numb is an adaptor protein, evolutionary conserved from flies to mammals, (13), which is involved in many cellular processes (14), and is able to behave as a cell fate determinant, being responsible for daughter cell polarization in asymmetric division (14–16). Numb has been described in cortical ventricular zone cells (17) and neural crest lineages (18), where it segregates preferentially in neural daughter cell during asymmetric division (16). Mammalian Numb is transcribed in four isoforms, namely p65, p66, p71, and p72, produced by alternative splicing (19), of which the p66 and p72 have been the main focus of research. Several studies described the role of Numb in stem cells compartment's maintenance, acting as intrinsic determinant through the interaction with signaling pathways such as Notch, p53 and Shh (20–23). To this regard, Numb was shown to control Gli1 function by inducing Gli1 ubiquitination and degradation (23, 24). Interestingly, Numb is abnormally expressed in many cancer types (25–29), and has been demonstrated to play a role in cancer stem cell subpopulation of colorectal cancer (30) and gliomas (31).

The role of Numb as fate determinant in different types of stem cells, the important role of Shh in the maintenance of NSCs and MB SLCs and the molecular relationship between Numb and Gli prompted us to investigate the role of Numb in the mouse cerebellar neural stem cells (NSCs) as well as in medulloblastoma stem-like cells (MB-SLCs). Moreover, we aimed to investigate the expression of Numb isoforms in MB subgroups, since previous studies were conducted before the definition of the molecular subgroups and also the differential expression of Numb isoforms was not explored (23).

## Materials and methods

### Cell cultures

Stem Cells. Cerebellar NSCs were obtained from cerebella of postnatal 4-day-old wild-type black 6 /C56 (C57BL/6) mice (Charles River). NSCs were derived as previously described (12).

Mouse Medulloblastoma stem-like cells (mMB-SLCs) were derived from spontaneous tumors arisen in Ptc+/– mice, as previously described (9, 32).

Human Medulloblastoma stem-like cells (hMB-SLCs) were derived from primary human MB during surgical resection as previously described (9). hMB-SLCs were immunostained with APC-conjugated anti-CD133 (Miltenyi Biotec) according to manufacturer's protocol and sorted using a FACSAriaIII (BD Biosciences) prior to experiments (32).

NSCs and MB-SLCs were cultured in Selective medium (SM) for stem cells enrichment, containing DMEM/F12 (Gibco) supplemented with 0.6% glucose, 25 mg/ml insulin, 60 mg/ml *N*-acetyl-L-cysteine, 2 mg/ml heparin, 20 ng/ml EGF, 20 ng/ml bFGF (Peprotech, Rocky Hill, NJ), 1X penicillin-streptomycin, and B27 supplement without vitamin A (Gibco).

For neurosphere/oncosphere forming assay NSCs and MB-SLCs were disaggregated to single cell and plated at clonal density (1–2 cells/mm^2^) into 96-well plates, in selective medium. After 10–14 days, the number of neurospheres or oncospheres was divided by the number of cells plated to determine the percentage of neurosphere forming cells and oncosphere forming cells, respectively.

To induce differentiation, NSCs were disaggregated and plated on poly-lysine coated dishes in differentiation medium containing platelet-derived growth factor (PDGF; 10 ng/ml) (Sigma, P3076), for 48 h (9).

Animal experiments were approved by local ethic authorities and conducted in accordance with Italian Governing Law (D.lgs 26/2014; Prot. no. 03/2013).

P19 were purchased from ATCC and maintained in Alpha Minimum Essential Medium with ribonucleosides and deoxyribonucleosides supplemented with 7.5% bovine calf serum, 2.5% fetal bovine serum, 2 mM l-glutamine, 100 U/ml penicillin, and 100 μg/ml streptomycin (Thermo scientific).

### Treatments

Transduced NSCs were treated with a Smoothened antagonist cyclopamine-KAAD, (Calbiochem), at the 1 μM, and with Smo-agonist SAG (200 nM, Alexis), for 48 h. For differentiation experiment, cells were treated with platelet derived growth factor (PDGF, Sigma-Aldrich) for 48 h.

### Immunofluorescence

To detect Gli1 and Numb, neurospheres were blandly disaggregated and plated on poly-lysine-coated Lab-Tek chamber slides (cover slips) for 2 h. Cells were fixed with 4% paraformaldehyde for 20 min at room temperature, incubated in blocking solution (5% normal goat serum, 1% BSA, 0.1% Triton X-100) and stained overnight with primary antibodies diluted in blocking solution and for 2 h with secondary antibodies. Primary antibodies were mouse anti-Gli1 and rabbit anti-Numb (Cell Signaling Technology Inc). 594- or 488-conjugated anti-mouse and anti- rabbit secondary antibodies were purchased from Molecular Probes (Invitrogen, Eugene, OR). Nuclei were counterstained with Hoechst reagent. Cover slips were mounted with fluorescence mounting medium (Dako, Carpinteria, CA). Images were acquired with Carl Zeiss microscope (Axio Observer Z1) and AxioVision Digital Image Processing Software.

### Lentiviral transduction

Neurospheres were transduced with pGreenZeo Lentiviral Reporter Vectors containing specific promoters for NANOG (Nanog-GFP) or CMV (Zeo-GFP) (9) and infected cells were selected with Zeocin (Thermo Fisher) treatment.

Numb p66 lentiviral infection was performed using a lentiviral vector pRRL–CPPT–CMV–PGK–GFP–WPRE (TWEEN) containing Numb p66 coding sequence (23). NSCs and MB-SLCs were infected for 48 h prior to analyses.

### Knockdown experiments

Silencing of endogenous Numb was performed using ON-TARGET plus Human NUMB siRNA (L-015902-00-0005) for hMB-SLCs and ON-TARGET plus Mouse Numb siRNA (L-046935-01-0005) for NSCs and mMB-SLCs. Dharmacon. Hiperfect reagent (Qiagen) was used for siRNA transfections according to manual instructions. After 72 h, cells were harvested and subjected to mRNA expression analysis.

### RNA extraction and gene expression analysis

Total RNA was isolated from cells and human tissue using Trireagent (Ambion) and reverse transcribed in cDNA as previously described (12). cDNA was used for quantitative RT-PCR (qRT-PCR) analysis using ViiA ™ 7 Real-Time PCR System and SensiFAST™ Probe Lo-ROX (Bioline).

For each mRNA analysis 10 ng of cDNA were used. We selected best coverage TaqMan gene expression assay from Applied Biosystems and used according to the manufacturer's instructions. To analyze Numb isoforms, the following assay IDs were used: murine Numb p66 Mm01302754_m1; murine Numb p72 Mm01304901_m1, human Numb p66 Hs01105435_m1; human Numb p72 Hs01105426_m1.

mRNA quantification was expressed in arbitrary units and each amplification reaction was performed in triplicate. All Results were evaluated using the 2-ΔΔCT method and values were normalized to three endogenous controls: ß-actin, ß2-microglobulin, and Gapdh.

### Western blot assay

Western blot was performed as previously described (33). Cellular pellets were lysed using lysis buffer: Tris-HCl pH 7.6 50 mM, deoxycholic acid sodium salt 0.5%, NaCl 140 mM, NP40 1%, EDTA 5 mM, NaF 100 mM, sodium pyrophosphate 2 mM, and protease inhibitors. Cellular lysates were separated on 8% acrylamide gel and western blot analysis was performed using standard procedures. Membranes were incubated overnight with the following antibodies: anti-Numb (ab4147; Abcam), anti-mouse Nanog (Cosmo Bio Co, Japan), anti-GAPDH (ab8245; Abcam), anti-Actin I-19 (sc-1616; Santa Cruz Biotechnology), anti-mouse Gli1 (#2643; Cell signaling), anti-NeuN (MAB377 Millipore), anti-βIII-tubulin (MAB 1637 Millipore). HRP-conjugated secondary antibodies (Santa Cruz Biotechnology) were applied on membranes and signals were visualized by enhanced chemiluminescence (ECL Advansta). Densitometry was performed using ImageJ software and protein levels were normalized to the respective loading control. Error bars represent mean ± standard deviation of at least three experiments.

### Human MB samples and controls

Surgical specimens of primary MBs were originated from a cohort of patients included in the present study, enrolled with Institutional Review Board approval, as previously described (34). Molecular subgroup classification was performed as described in (34). Number of MBs analyzed for each molecular subgroup: WNT n: 10; SHH n: 25; G3 n: 25; G4 n: 19.

Correlation analysis between the isoforms was measured using GraphPad Prism 6 software (La Jolla, CA, USA).

Commercial non-neoplastic cerebellum was purchased from Bio-chain Institute (*n* = 4: R1234039-50, Total RNA-Human Brain cerebellum Adult; *n* = 4: R1244041-50 and R1244040-50, Total RNA-Human Brain cerebellum Fetal).

## Results

### Numb has a pro-differentiation role in cerebellar neural stem cell (NSCs)

**Figure 1:**
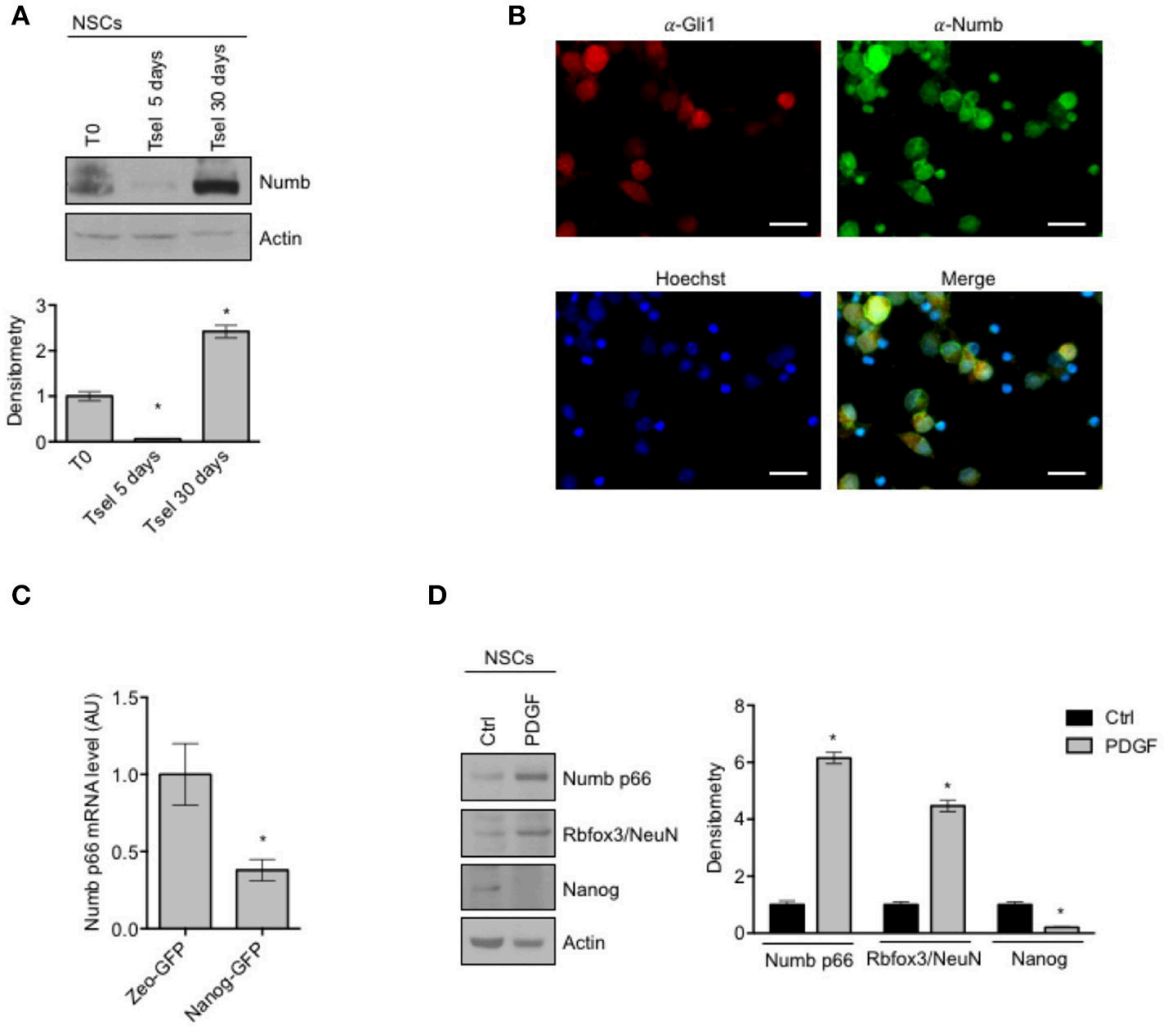
Numb expression in NSCs. **(A)** Representative Western Blot (WB) (up) and densitometric analysis (bottom) of endogenous Numb isoforms (p66 and p72) in NSCs grown in selective medium for 5 or 30 days, compared to bulk cells (T0). Actin was used as loading control. *P*-values *p < 0.05. **(B)** Representative images of immunofluorescence staining of NSCs for Numb (green) and Gli1 (red); nuclei are counterstained with Hoechst (blue). Scale bar: 10 μm for all panels. **(C)** qRT-PCR analysis showed mRNA expression of Numb p66, evaluated in NSCs infected with lentivirus carrying Nanog-GFP or Zeo-GFP as control and sorted for GFP. Data represent means ± SD from three independent experiments. *P*-values *p < 0.05. **(D)** Representative Western Blot (WB) (left) and densitometric analysis (right) of NSCs before and after *in vitro* differentiation for 48 h (PDGF); p66, Rbfox3/NeuN, Nanog were evaluated. Actin was used as loading control. *P*-values *p < 0.05. **(A–D)** Data are means ± SD from three independent experiments. Full-length images are presented in Supplementary Figures.

Involvement of Numb in cell determination and differentiation and in cortical neurogenesis has already been described (35), while the role of Numb in cerebellar neural stem cell (NSCs) differentiation has not been studied to date. First of all, we evaluated Numb protein expression in NSCs with respect to starting population (Figure 1A). NSCs were identified as the neurosphere forming cells after at least 30 days in selective medium (SM), and were compared to both the bulk cell population and to cerebellar cells after 5 days in SM. Notably, Numb protein level was lower at day 5 in SM with respect to both bulk population and NSCs, probably due to a selection of stem cells in medium, and its expression increased at day 30, when NSC culture was established (Figure 1A). Since only one band was revealed by western blot analysis, we compared Numb protein expression pattern of NSCs with the protein expression in murine embryonal carcinoma P19 cells after differentiation stimuli. P19 cells represent a model of neuronal differentiation which express both Numb p66 and Numb p72 isoforms (19). Interestingly, NSCs expressed high levels of the Numb p66 isoform while Numb p72 was not detectable (Supplementary Figure 1). To investigate the distribution of Numb positive cells in the heterogeneous population of neurosphere culture, we performed immunofluorescence staining of Numb and Gli1 (Figure 1B), a stemness marker in the context of cerebellar NSCs (9). Interestingly, Numb is expressed in both Gli1 positive and Gli1 negative cells.

To further investigate whether Numb was associated with stemness features in the neurosphere population, we sorted cells according to their expression of the stemness factor Nanog (9), and we observed that Nanog positive cells expressed significantly lower levels of Numb p66, with respect to control (Figure 1C).

In order to explore the role of Numb in influencing the balance between stemness and neural differentiation, we evaluated Numb p66 protein level with western blot analysis, in NSCs before and after *in vitro* differentiation (Figure 1D). Numb p66 protein level was increased in NSCs after differentiation stimuli such as platelet-derived growth factor (PDGF), together with an enhanced expression of differentiation markers (Rbfox3/NeuN) and a reduced expression of Nanog stemness marker.

**Figure 2:**
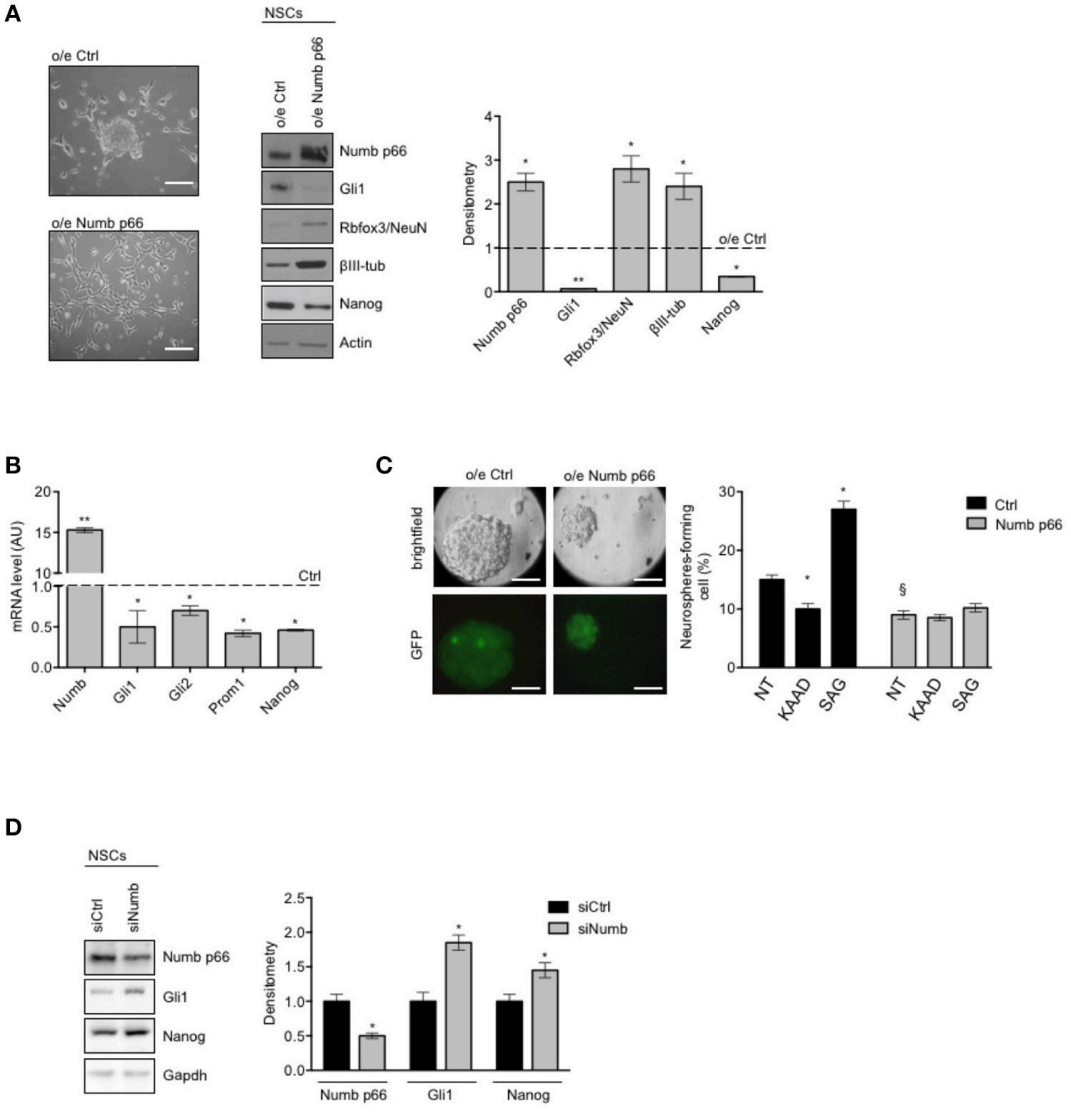
Numb antagonizes stemness and promotes differentiation of cerebellar NSCs. **(A)** (Left) Representative bright-field images of neurospheres after overexpression of p66 (or Ctrl). Scale bar: 100 μm. Representative western blot images (center) and densitometric analysis (right) of analysis of p66, stemness, and/or Hedgehog markers Nanog and Gli1 and differentiation markers Rbfox3/NeuN and βIII-tubulin (βIII-tub), in NSCs after overexpression of p66 vs. Ctrl. Actin was used as loading control. *P*-values *p < 0.05. **(B)** mRNA expression levels of Numb p66 and stemness and/or Hedgehog markers (Gli1, Gli2, Prom1, and Nanog), in NSCs after overexpression of p66. Dashed bars represent control cells (Ctrl). Data are means ± SD from three independent experiments. *P*-values *p < 0.05; **p < 0.01. **(C)** (Left) LvNumb-transduced NSCs were plated for clonogenic assay as described in Material and Methods. Upper panels show brightfield images of secondary neurospheres, lower panels show GFP of transduced cells. Scale bar: 100 μm. (Right) Neurosphere-formation capacity of LvNumb-transduced NSCs and treated with cyclopamine-KAAD (KAAD) to suppress endogenous Hedgehog signaling or with Smo-agonist (SAG). Data are means ± SD from three independent experiments. *P-values (vs. NT), *p < 0.05. §*P*-value (vs. LvCtrl), §*p* < 0.05. **(D)** Representative Western Blot (WB) (left) and densitometric analysis (right) of NSCs before and after *in vitro* differentiation for 48 h (PDGF); p66, Rbfox3/NeuN, Nanog were evaluated. Actin was used as loading control. *P*-values *p < 0.05. GAPDH was used as loading control. **(A–D)** Data are means ± SD from three independent experiments. Full-length images are presented in Supplementary Figures.

We next proceeded to investigate the role of Numb p66 in NSCs by modulating its expression. We performed lentiviral infection of NSCs with a virus encoding the ORF of Numb p66 (LvNumb) and evaluated the effects after 48 h. LvNumb-transduced NSC cells showed a differentiated phenotype with adherent morphology (Figure 2A, left), up-regulation of differentiation neural markers (Rbfox3/NeuN and βIII-tubulin) and down-regulation of the stemness markers Nanog and Gli1 (Figure 2A, right). Thus, our data support the role for Numb in promoting the “differentiated phenotype” of NSCs.

To deepen our understanding of the relationship between Numb p66 expression, stemness features and Shh signaling, we evaluated the expression of relevant markers in LvNumb-transduced NSCs. In detail, we observed a strong reduction, at transcription level, of both key components in the Shh pathway (Gli1, Gli2) and the stemness markers Prom1 and Nanog compared with control cells (Figure 2B). Consistently with mRNA level data of stemness markers, Numb overexpression also impaired the ability of NSCs to form secondary neurospheres, i.e., their clonogenicity (Figure 2C) and Numb transduced NSCs formed smaller neurospheres. We previously showed that the modulation of the Shh signaling in NSCs is able to significantly enhance or impair clonogenicity (9). Interestingly, after modulation of the Shh signaling in LvNumb-transduced NSCs, we did not observe any significant variation of self-renewal, suggesting that Numb overexpression could counteract this pathway (Figure 2C, right).

In order to further investigate the role of Numb p66 in NSCs, we performed silencing of Numb (siNumb) and observed an upregulation of Nanog and Gli1 (Figure 2D). Altogether, these data support a role for Numb p66 in NSCs where it induces neural differentiation and controls stemness by negatively regulating the Shh signaling.

### NUMB p66 controls self-renewal of SHH MB stem-like cells

To investigate the role of NUMB in cancer stem-like cells (SLCs) from SHH MB, we performed the following set of experiments in SLCs derived from a human SHH MB, referred to as hMB-SLCs, as previously described (32). First of all, we sorted hMB-SLCs for the stemness marker CD133 (32), and analyzed the expression profile of CD133 positive (CD133+) and negative (CD133-) cells.

**Figure 3:**
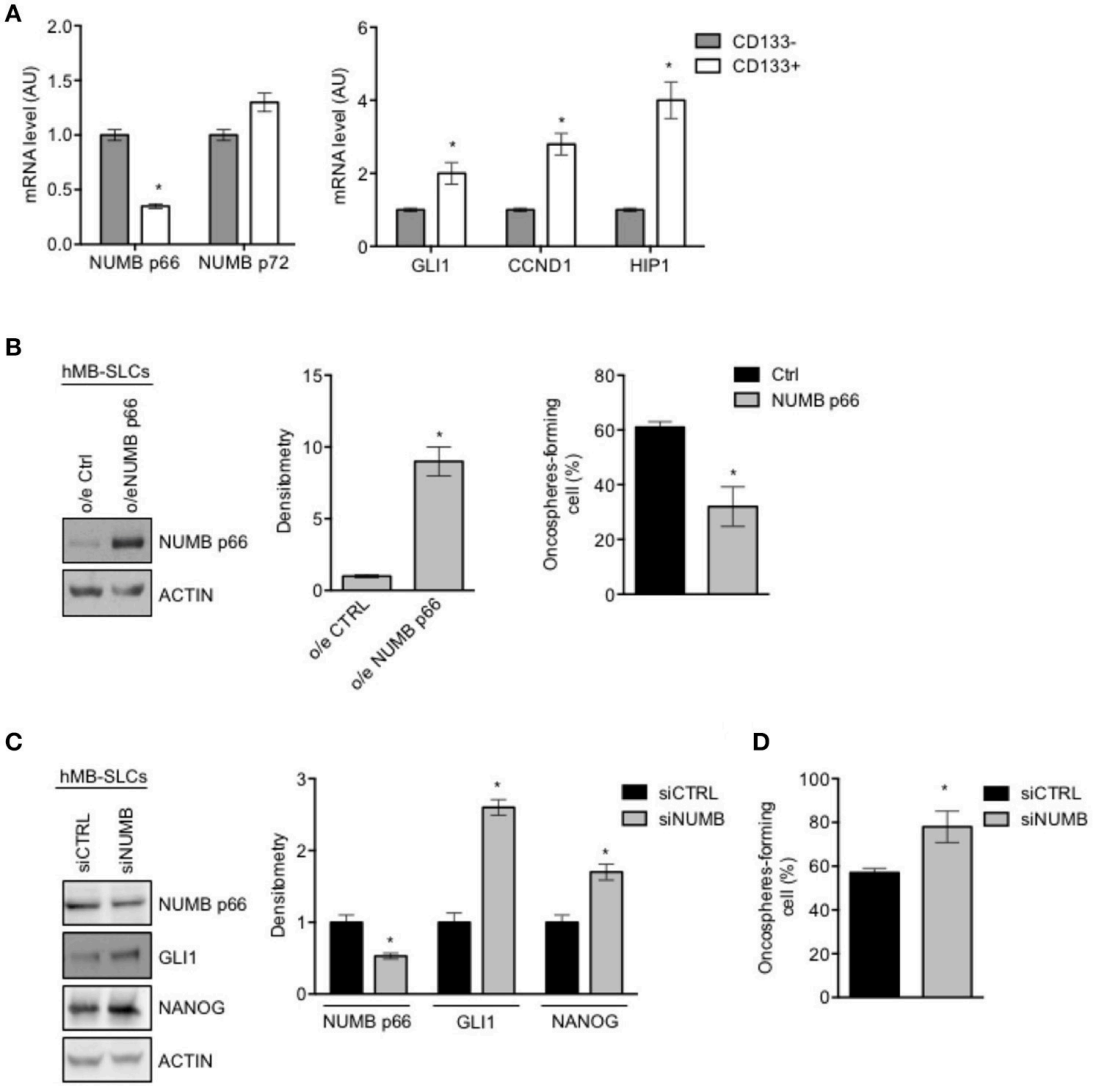
Modulation of NUMB in hMB-SLCs. **(A)** qRT-PCR analysis showed mRNA expression of NUMB isoforms, p66, and p72, and of Hedgehog markers (GLI1, CCND1, HIP1) evaluated in hMB-SLCs CD133^+^ vs. CD133^−^. Data represent means ± SD from 3 independent experiments. *P*-values *p < 0.05. **(B)** Representative western blot images (left) and densitometric analysis (center) of p66 in hMB-SLCs, after overexpression of p66 vs. Ctrl. ACTIN was used as loading control. (Right) Oncospheres-formation capacity of LvNumb-transduced hMB-SLCs vs. LvCtrl. *P*-values *p < 0.05. **(C)** Representative images of western blot (left) and densitometric analysis (right) analysis of p66, NANOG, and GLI1, in hMB-SLCs transfected with siRNA against NUMB (siNUMB) or non-targeting siRNA controls (siCTRL). ACTIN was used as loading control. *P*-values *p < 0.05. **(D)** Oncospheres-formation capacity of siNUMB hMB-SLCs vs. siCTRL. *P*-values vs. siCTRL. Data represent means ± SD from three independent experiments. *P*-values *p < 0.05. **(B–C)** Data are means ± SD from three independent experiments. Full-length images are presented in Supplementary Figures.

As shown in Figure 3A, expression level of NUMB p66 was significantly lower in CD133+ cells, with respect to CD133– ones, while NUMB p72 showed a positive trend in CD133 positive cells, without reaching statistical significance. Interestingly, mRNA levels of NUMB p66 resulted higher than NUMB p72 in CD133+ hMB-SLCs (Supplementary Figure 1B), in accordance with protein data. We also investigated markers of the Shh pathway (GLI1, CYCLIN D1, HIP1), that we previously showed to drive stemness in cerebellar NSCs and SHH MB SLCs (9). Shh pathway resulted more active in CD133+ (Figure 3A). These data suggest that NUMB p66 was associated with a reduced state of stemness hMB-SLCs.

We then proceeded to evaluate the role of NUMB in hMB-SLCs *in vitro*. As shown in Supplementary Figure 1A, hMB-SLCs express high protein levels of NUMB p66 isoform. To explore the role of NUMB p66 in hMB-SLCs, we performed lentivirus-mediated overexpression of NUMB p66 (Figure 3B, left). We then evaluated the clonogenicity of LvNumb-transduced hMB-SLCs cells, a significant decrease of the oncospheres' forming capability was observed in hMB-SLCs overexpressing NUMB vs. control cells (Figure 3B, right).

To further understand the role of p66 in regulating stemness in MB-SLCs, we depleted the expression of NUMB using small interfering RNA (siNUMB) in hMB-SLCs. As shown in Figure 2C, following NUMB silencing, hMB-SLCs showed an increase of GLI1 protein level respect to control siRNA-transfected cells. Moreover, siNUMB hMB-SLCs showed an increase of NANOG stemness marker (Figure 3C) and oncosphere-forming ability (Figure 3D). These results suggest that NUMB may have a role in negatively regulating stemness features in hMB-SLCs. Additionally, we performed silencing of Numb in stem like cells isolated from murine SHH MB spontaneously arisen in Ptch +/− mice (9, 32). Numb silencing caused an increase in Gli1 and Nanog protein levels (Supplementary Figure 2A) and enhanced clonogenicity (Supplementary Figure 2B).

Our evidence show that NUMB plays a critical role in influencing MB-SLCs behavior, blocking the Sonic Hedgehog signaling and stemness features.

### Numb alternative splicing in medulloblastoma

We then investigated NUMB expression in a cohort of MB using the MB dataset provided by Cavalli (Tumor Medulloblastoma–Cavalli−763 -rma_sketch—hugene11t) in the R2 platform (36). MB samples were divided into molecular subgroups and according to total NUMB expression level (low or high). As shown in Supplementary Figure 3, low expression of NUMB was associated with shorter overall survival (OS) in SHH and G3 subgroups.

Unfortunately, in the publicly available datasets the present probes do not discriminate the different isoforms of NUMB.

The expression level of NUMB 66 (p66) [Isoform 2, identifier: P49757-2] and NUMB 72 (p72) [Isoform 1, identifier: P49757-1] was evaluated in a cohort of human MB patients' samples characterized and divided in molecular subgroups.

**Figure 4:**
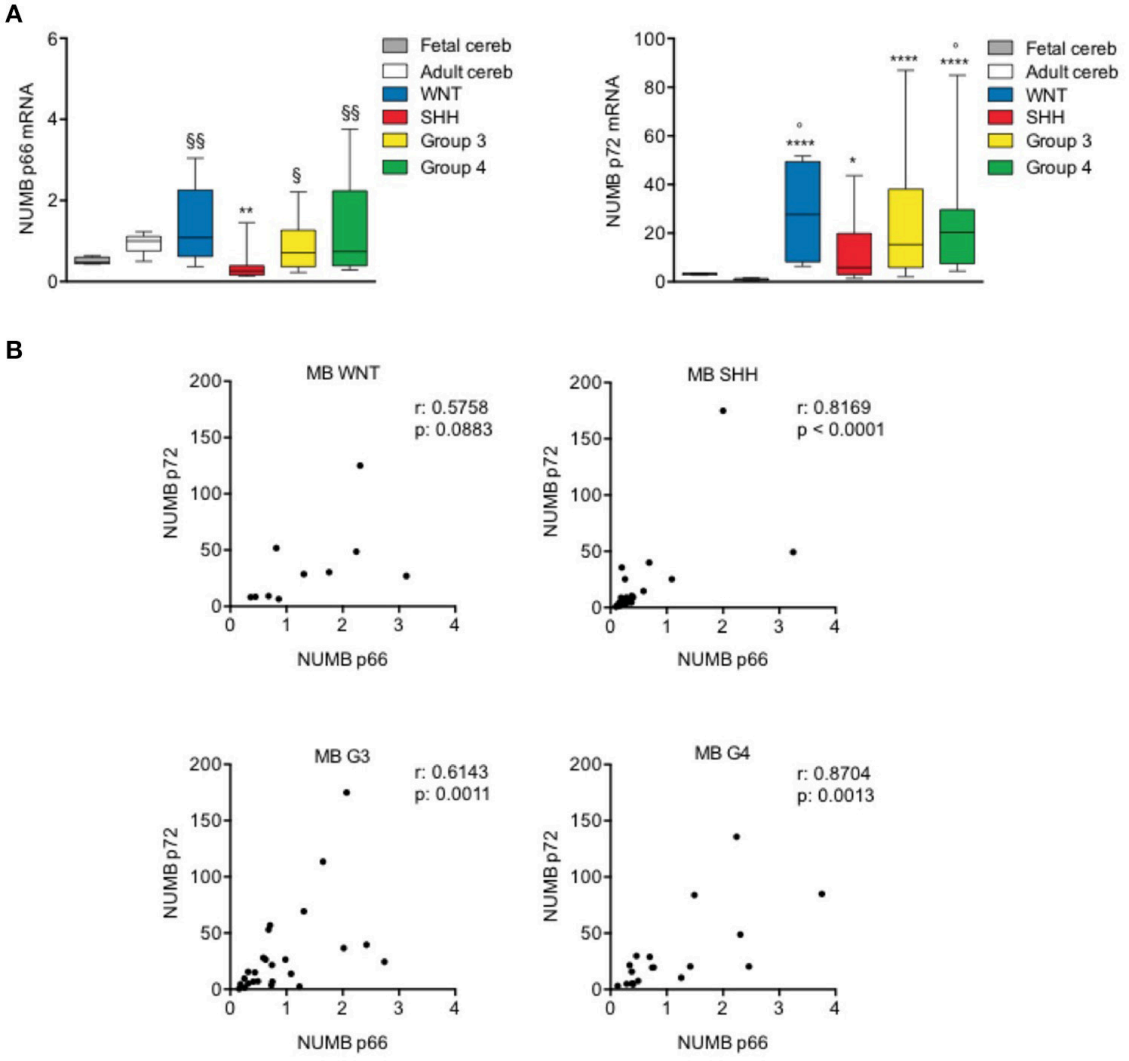
NUMB isoforms expression in human MB samples. **(A)** qRT-PCR analysis showed NUMB isoforms [p66 (left) and p72 (right)] mRNA expression for molecular subgroups of primary MBs (WNT *n*: 10; SHH *n*: 25; G3 *n*: 25; G4 *n*: 19), adult normal cerebellum (Adult cereb) and fetal normal cerebellum (Fetal cereb). *P-value vs. Adult cereb, *p < 0.05; **p < 0.01; ****p < 0.0001. ° = *P* vs. Fetal cereb, °*p* < 0.05. ^§^*P*-value vs. SHH MBs, ^§^*p* < 0.05; ^§§^*p* < 0.01. **(B)** Pearson's correlation test of p66 vs. p72, in human MB subgroups, to evaluate the link between the expression of NUMB isoforms (r = correlation coefficient; *p* < 0.05 significance).

The transcript level of p66 resulted significantly reduced only in SHH subgroup, whereas the p72 mRNA level was significantly up-regulated in all tumor molecular subgroups, as shown in Figure 4A, compared with normal adult cerebellar tissue (control). Interestingly, the up-regulation of p72 was stronger and more significant in G3, G4, and WNT with respect to SHH, although not reaching statistical significance in the comparison among subgroups. Furthermore, the expression of Numb p66 transcript was significantly higher in non-SHH tumors with respect to SHH. We also investigated the mRNA expression of NUMB isoforms in normal fetal cerebellum. With respect to adult tissues, fetal cerebellum showed a trend toward down-regulation of p66 and a trend of up-regulation of p72, even though it didn't reach statistical significance (Figure 4A). NUMB p66 expression in MBs failed to show statistical significance in the comparison with fetal cerebellum, and NUMB p72 resulted statistically up-regulated only in G4 MBs and WNT MBs. These data support previous findings that showed that SHH MB derive from of proliferating granule cell progenitors in the external granular layer (EGL) (37), that physiologically dissipates during the first year of postnatal life (38).

Next, we performed a correlation analysis of the expression level of the two isoforms analyzed that indicated significant positive correlation (*p* < 0.05) in SHH, G3, and G4 subgroups. WNT subgroup showed a trend toward positive correlation but didn't reach the statistical significance, possibly due to the small number of samples included in the study, with respect to other subgroups (Figure 4B).

Together, these data suggest a different role of NUMB p66 and p72 isoforms in human MB samples. Interestingly, we demonstrated a decreased expression of p66 only in the SHH subset, suggesting that different isoforms may exert divergent functions in the different MB subgroups.

## Discussion

In this study we investigated for the first time, the expression of the two main NUMB isoforms p66 and p72 in a wide MB cohort, finding that different NUMB isoforms were differentially expressed among subgroups.

Indeed, NUMB p66 and p72 have been shown to have different/opposite roles regulating cellular functions. In murine embryonic carcinoma cells, it is described that p66 isoform is involved in differentiation but not in proliferation, whereas p72 has a role in proliferation but not in differentiation (39). This difference in the role of each isoform could explain the different functions described for NUMB in different types of cancer.

Specifically, NUMB has been described as an oncosuppressor in breast cancer (29), esophageal squamous cell carcinoma (27) and mesothelioma (40), but evidence showed also a role for NUMB as an oncogene in hepatocellular carcinoma (41), in astrocytomas (42) in cervical squamous carcinoma cells (43), and in endometrial cancer (44).

These different roles could reflect the different isoforms' expression in our cohort of human MB samples. Indeed, regardless of the molecular subgroup, NUMB p72 is up-regulated. The expression of this isoform could have a role in accelerating cell proliferation and promoting EMT features in MB as suggested by studies in other context (41, 45).

Interestingly, we found that NUMB p66 was significantly down-regulated in SHH MB only. Numb p66 is expressed during development and in adult brain (39, 46), and in murine P19 embryonic carcinoma cell line, p66 is reported to promote neural differentiation (39). Numb p66 was also shown to be down-regulated in murine models of SHH MB and to control Shh pathway activation through the regulation of Gli1 function, via its ubiquitination and proteasome dependent degradation (23, 24). Thus, our results strongly suggest that low levels of NUMB p66 in SHH MB contribute to Shh pathway deregulation keeping cancer cells in an undifferentiated state and enhancing their cancer stemness features. Altogether these results point out to different roles for NUMB isoforms in the MB subgroups possibly reflecting the diverse cell signaling pathways governing them. We believe that the dual role of NUMB as oncosuppressor or oncogene might be ascribed to the different isoform expressed, a topic that has not been fully investigated in the cited literature.

NUMB isoforms differential expression is controlled by alternative splicing. Of note, RBFOX3 a member of RNA-binding Fox (Rbfox) family, is one of the most important regulators of NUMB alternative splicing in neuronal lineages (47). RBFOX3/NEUN is expressed in neurons (47, 48) and during spinal neuronal development, RBFOX3/NEUN regulates neuronal differentiation promoting the alternative splicing of NUMB mRNA, from the isoform p72 to p66 (47). Interestingly, we found up-regulated RBFOX3/NEUN both after *in vitro* differentiation of NSCs (Figure 1D) and after Numb p66 over-expression (Figure 2A), suggesting that there could be a regulatory feedback between Numb p66 and RBFOX3/NEUN. As shown in Figure 1D, RBFOX3/NEUN is up-regulated during differentiation of NSCs along with Numb. Moreover, SHH MB shows the highest expression of RBFOX3/NEUN compared to the other molecular subgroups (Supplementary Figure 4). These evidence suggest that RBFOX3/NEUN could be responsible for NUMB alternative splicing in SHH MB context, leading to isoforms shift from p72 to p66.

Notably, we identified expression of Numb p66 also in NSCs and in MB-SLCs, as a common feature of “stem cell phenotype.” We have previously demonstrated that these cellular models are characterized by Hedgehog pathway activation (9, 49). Numb p66 expression inversely correlates with stem cells features: indeed, NSCs and SHH MB-SLCs show high clonogenic potential when Numb is silenced (Figures 2C, 3C), where it cannot antagonize survival pathways, such as Hedgehog signaling (23). We demonstrated the role of the Numb p66 isoform in promoting neural differentiation, antagonizing the expression of stemness/Shh markers and inhibiting self-renewal of stem cells.

In conclusion, in this study we demonstrated for the first time the expression of Numb isoforms in Medulloblastoma, highlighting possible different roles for each isoform in MB subgroups, that we believe are worthy of further investigation in follow up studies. Moreover, we described the suppressive role of Numb p66 on stemness features of cerebellar NSC and of SHH MB-SLCs.

## Author contributions

LA and AP designed experiments, analyzed the data, and wrote the manuscript. AM, EM, AC, ZB, and MM performed the experiments and analyzed the data. EDS, FG, FL, and EF assisted the study.

### Conflict of interest statement

The authors declare that the research was conducted in the absence of any commercial or financial relationships that could be construed as a potential conflict of interest.

## Abbreviations

MB: Medulloblastoma
Shh: Sonic Hedgehog
NSC: Neural Stem Cells
SLC: Stem-like cells
LvNumb: Lentivirus Numb
hMB-SLC: Human Medulloblastoma stem-like cells
MB-SLC: Murine Medulloblastoma stem-like cells.
