# Supplementary figures and images for: Numb Isoforms Deregulation in Medulloblastoma and Role of p66 Isoform in Cancer and Neural Stem Cells
Source: Front Pediatr. 2018 Nov 1;6:315. doi: 10.3389/fped.2018.00315 (PMC6221942; doi:10.3389/fped.2018.00315)

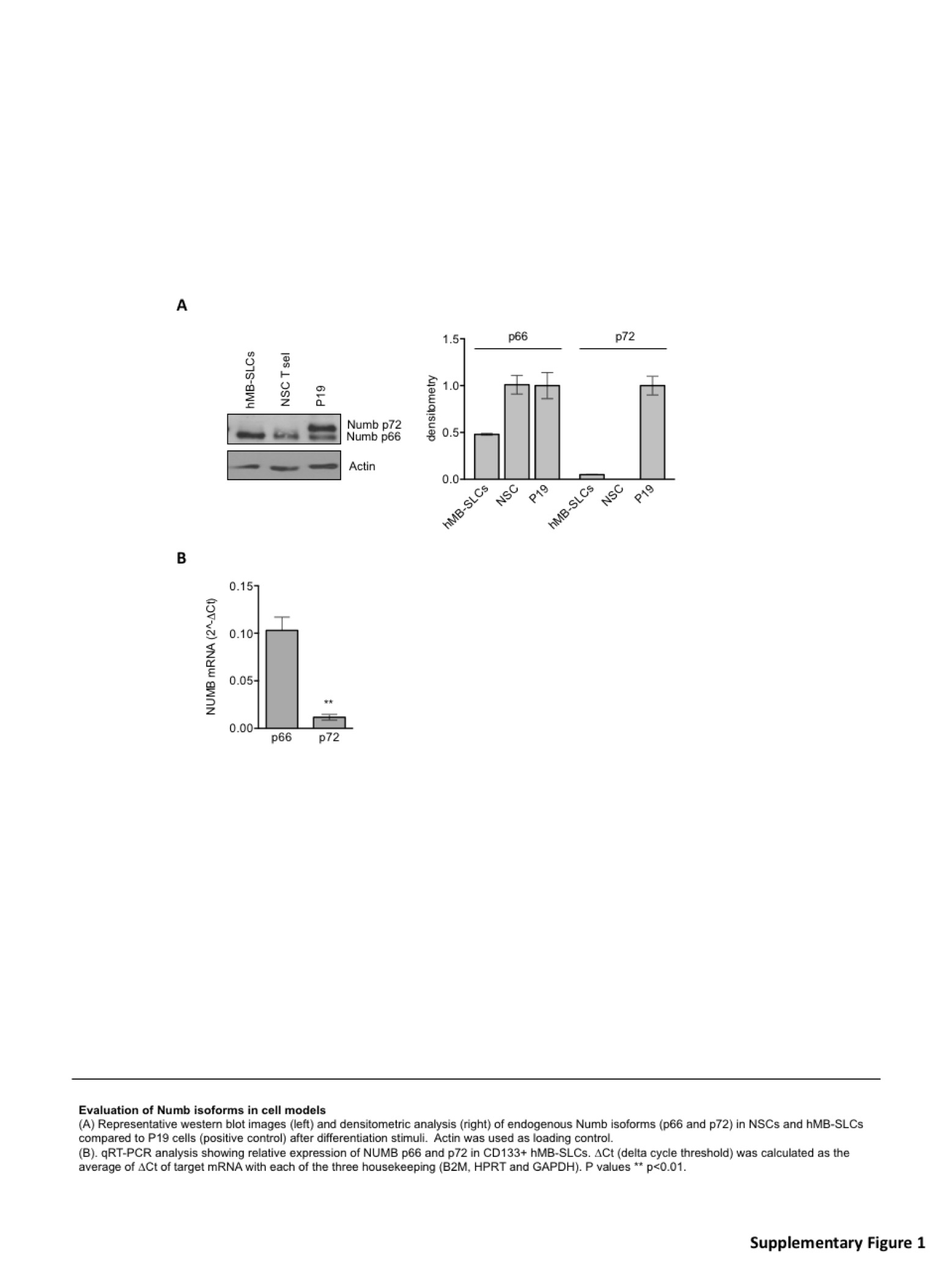

Supplement: Supplementary file 1 [file Image_1.JPEG]

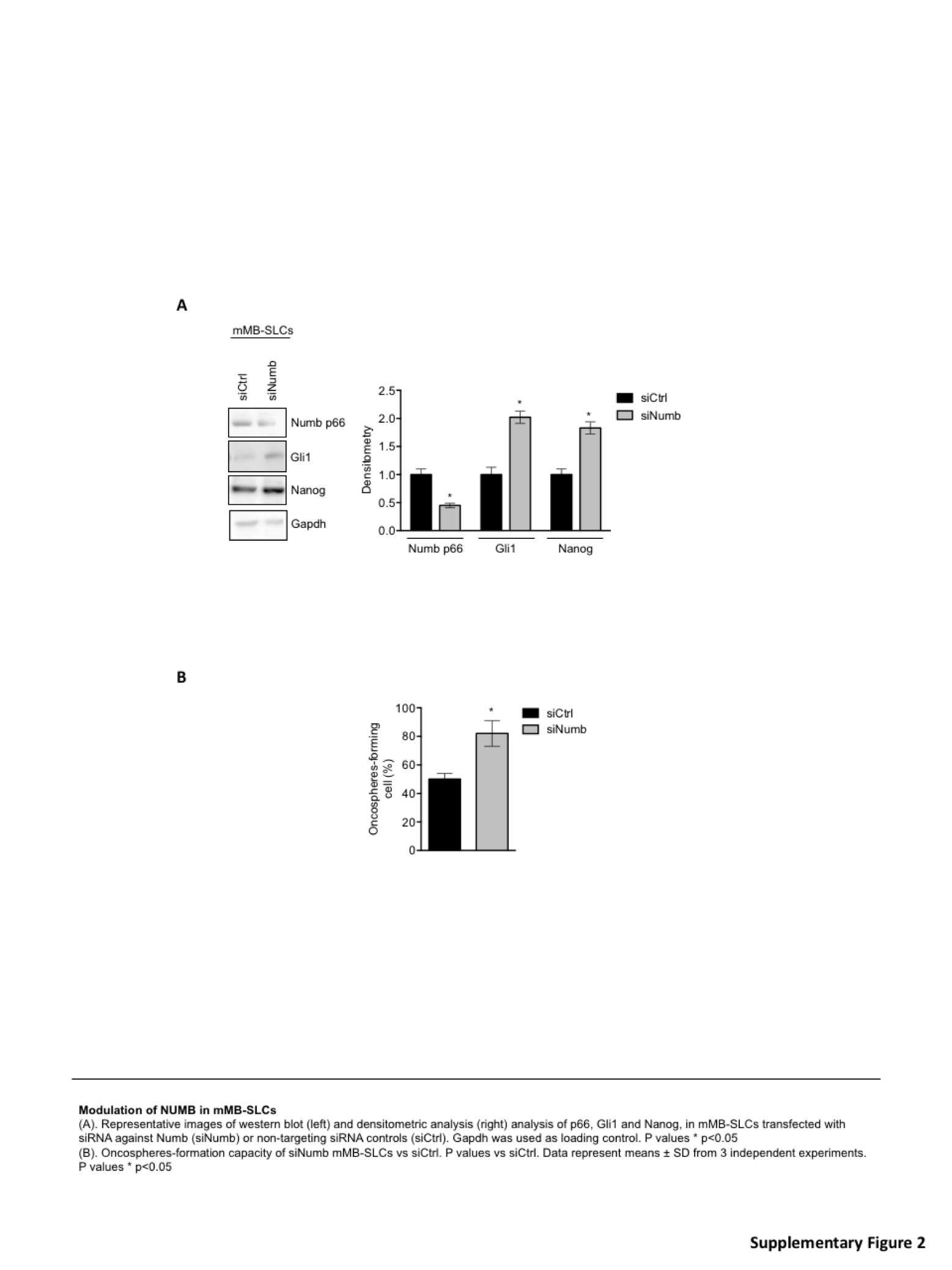

Supplement: Supplementary file 2 [file Image_2.JPEG]

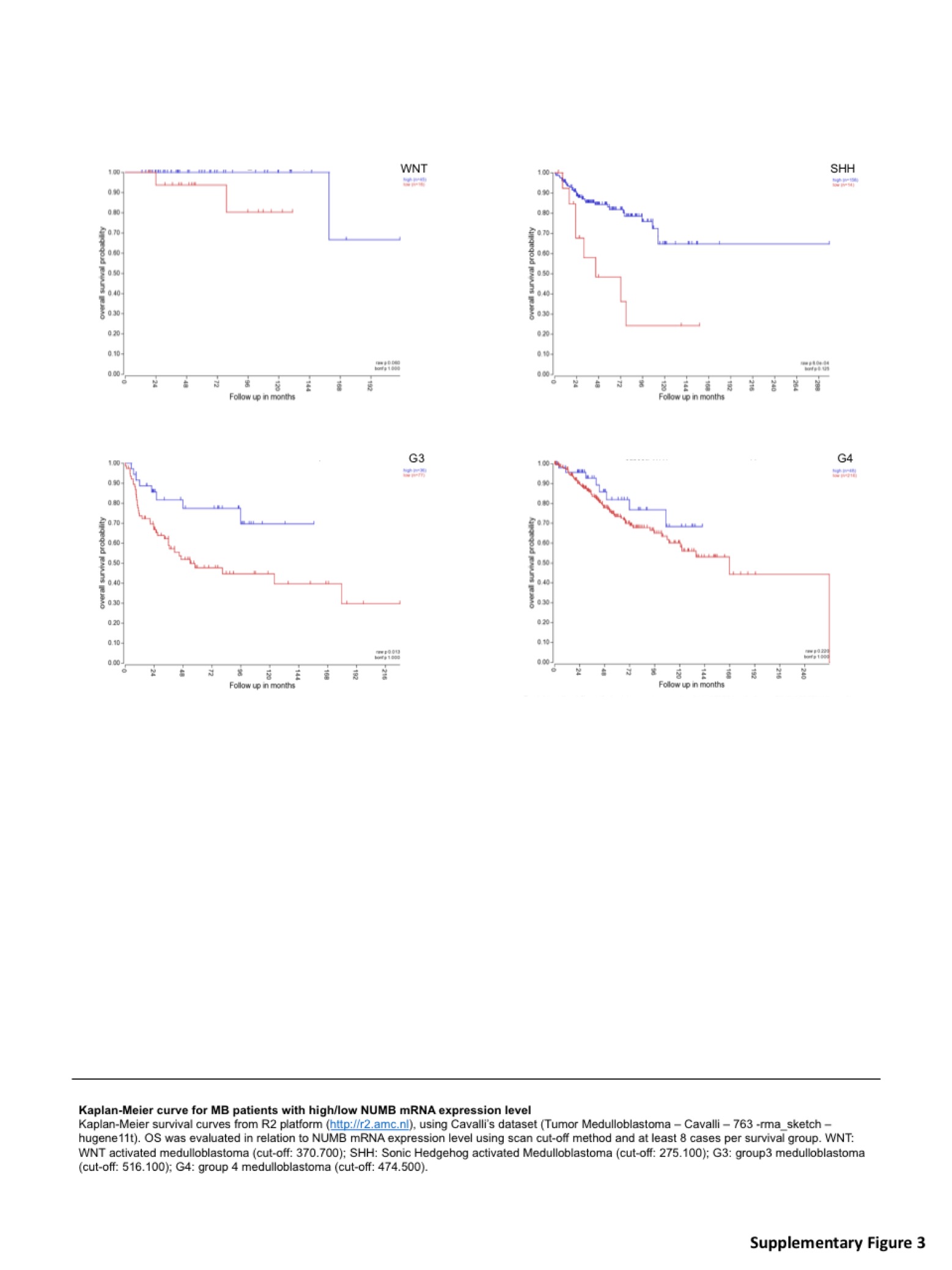

Supplement: Supplementary file 3 [file Image_3.JPEG]

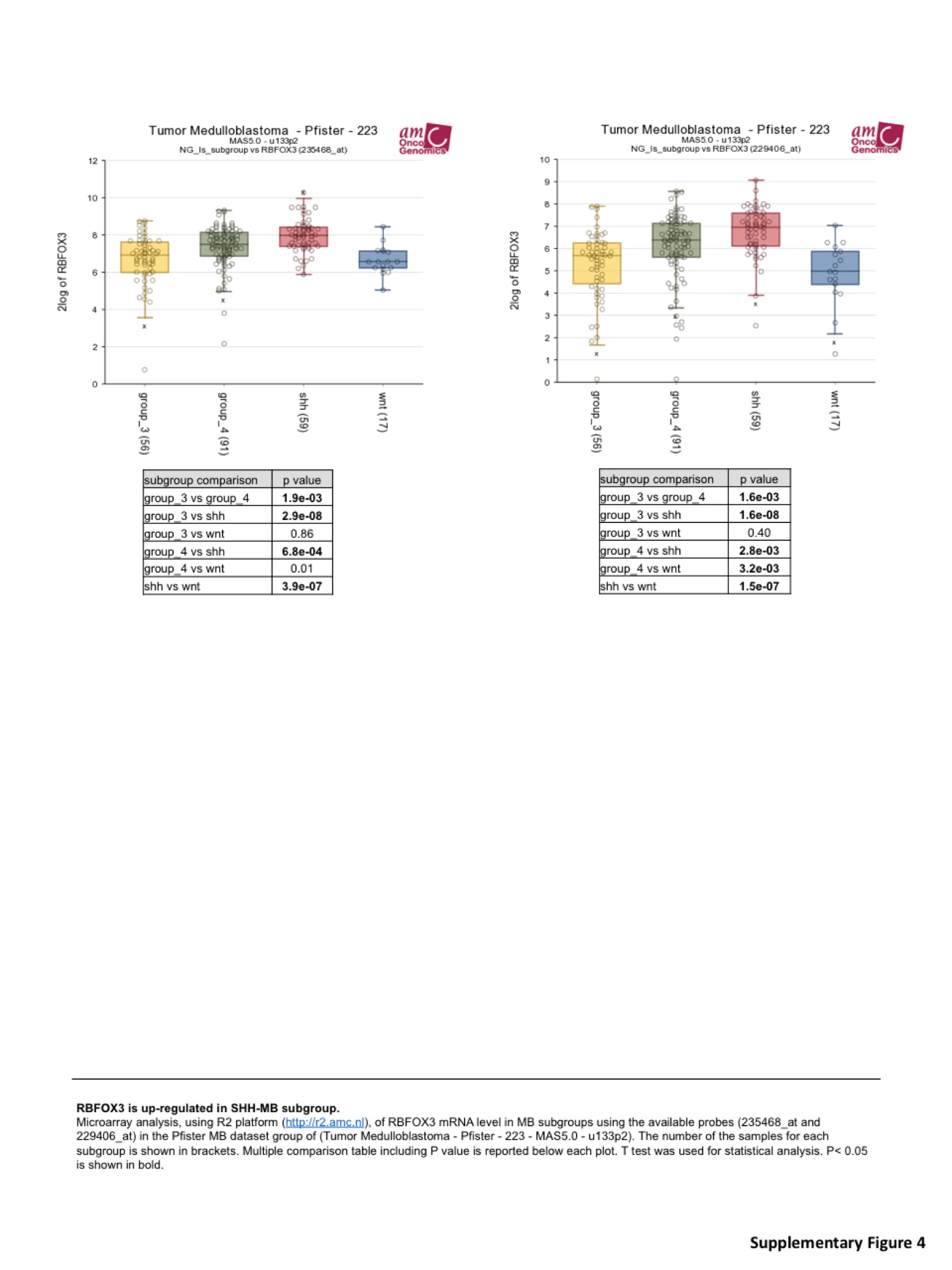

Supplement: Supplementary file 4 [file Image_4.JPEG]

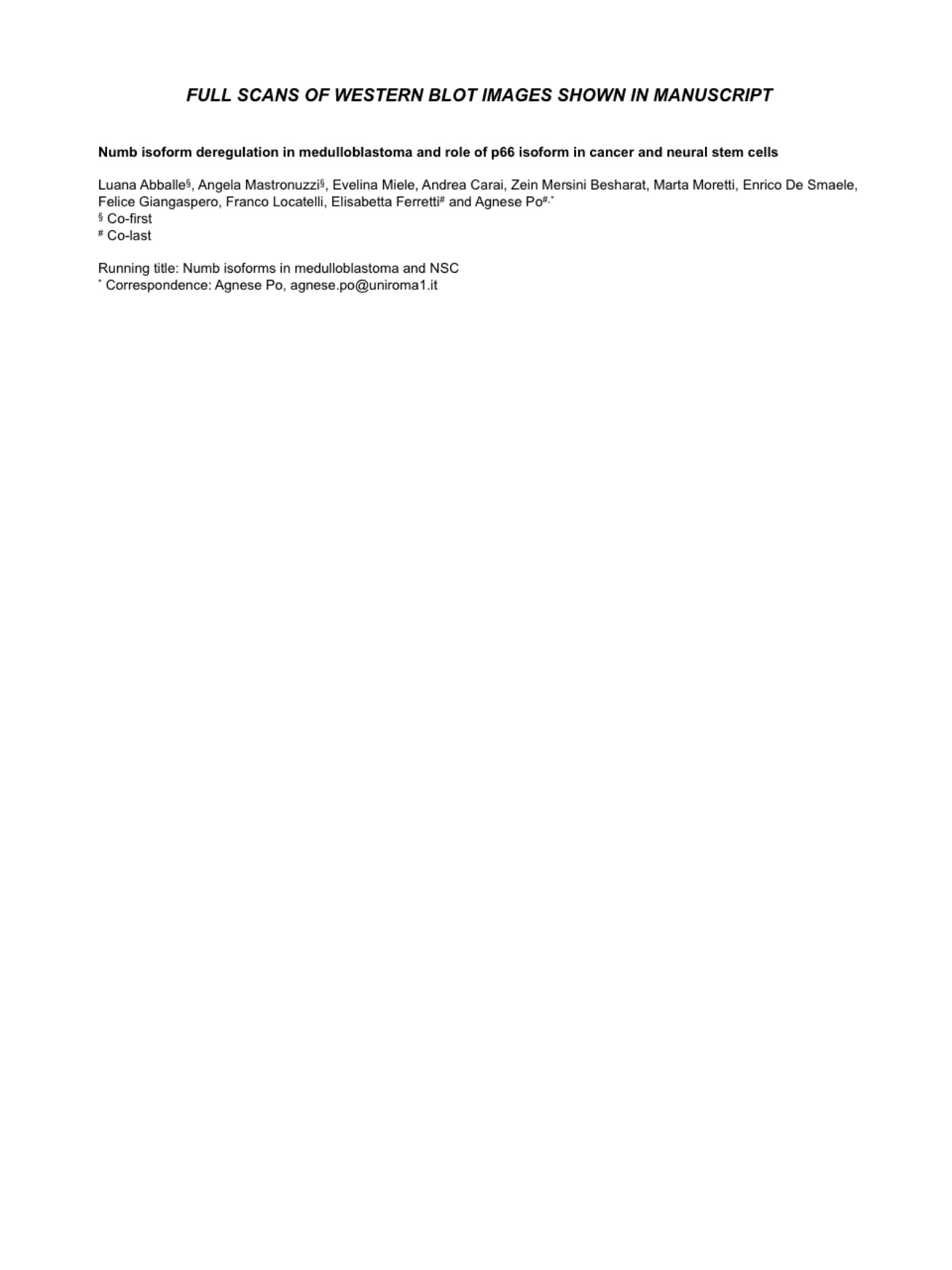

Supplement: Supplementary file 5 [file Image_5.JPEG]

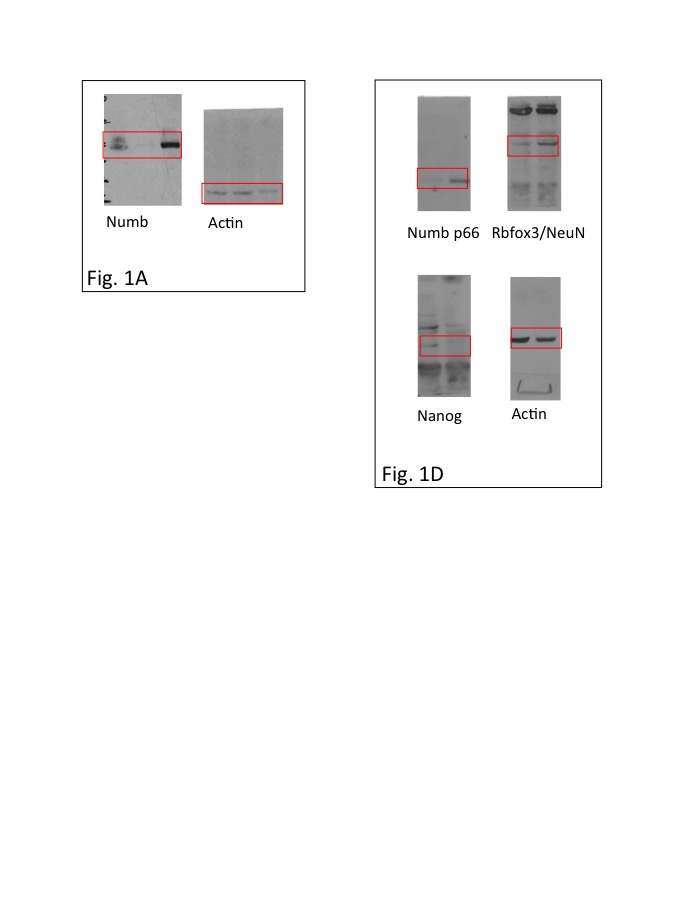

Supplement: Supplementary file 6 [file Image_6.jpg]

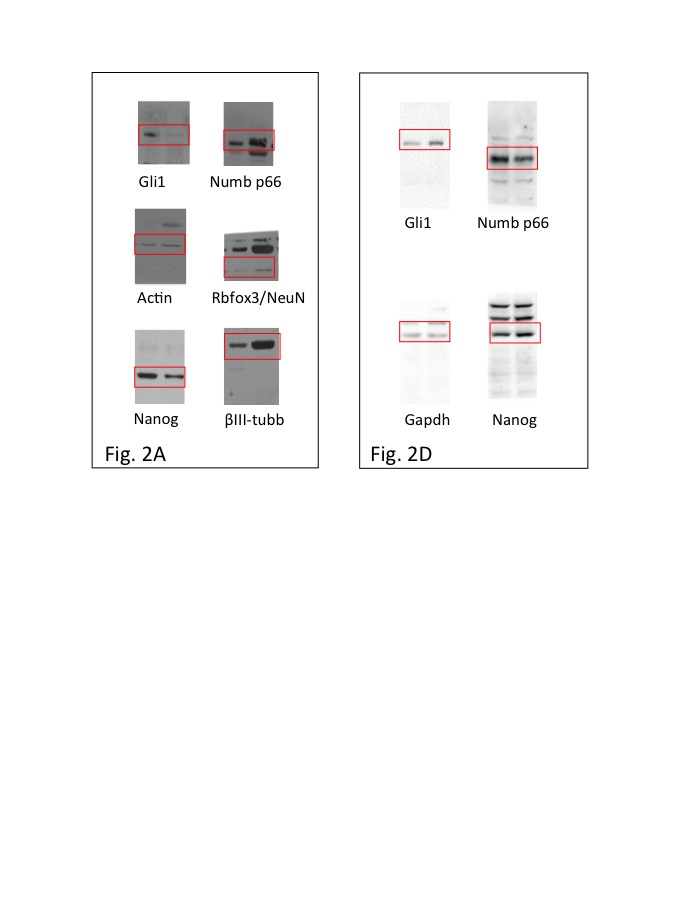

Supplement: Supplementary file 7 [file Image_7.jpg]

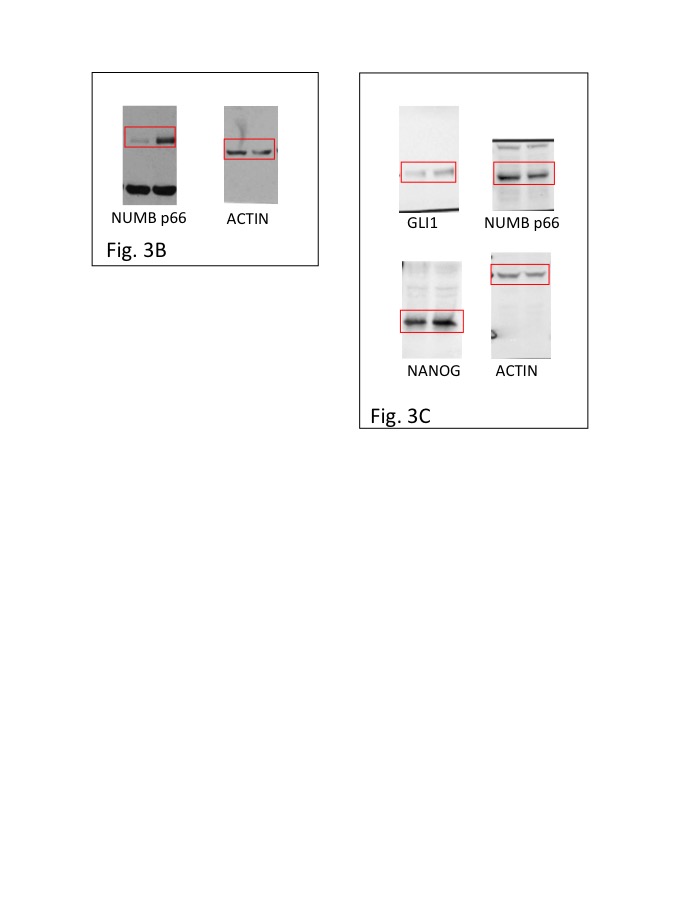

Supplement: Supplementary file 8 [file Image_8.jpg]

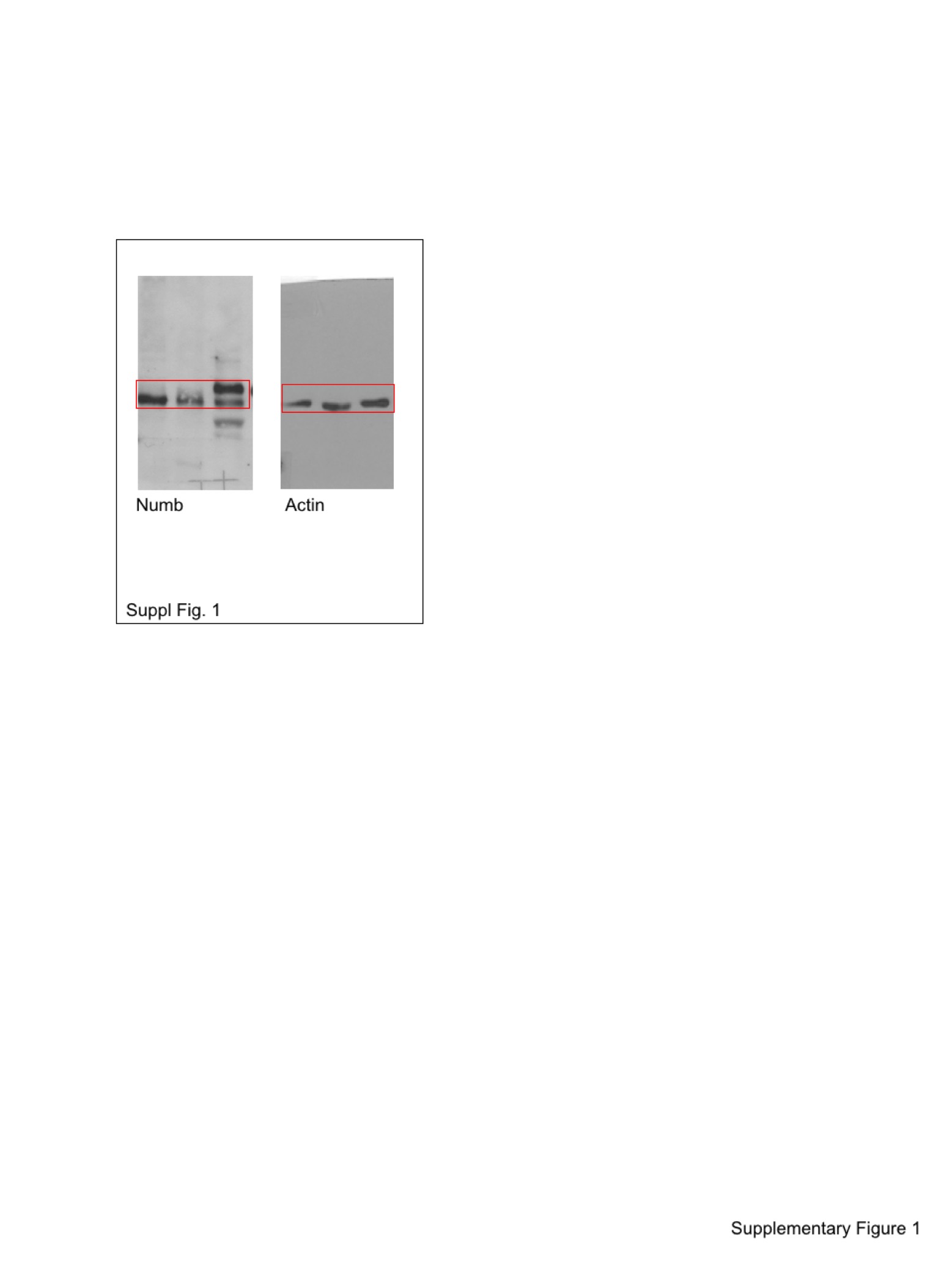

Supplement: Supplementary file 9 [file Image_9.JPEG]

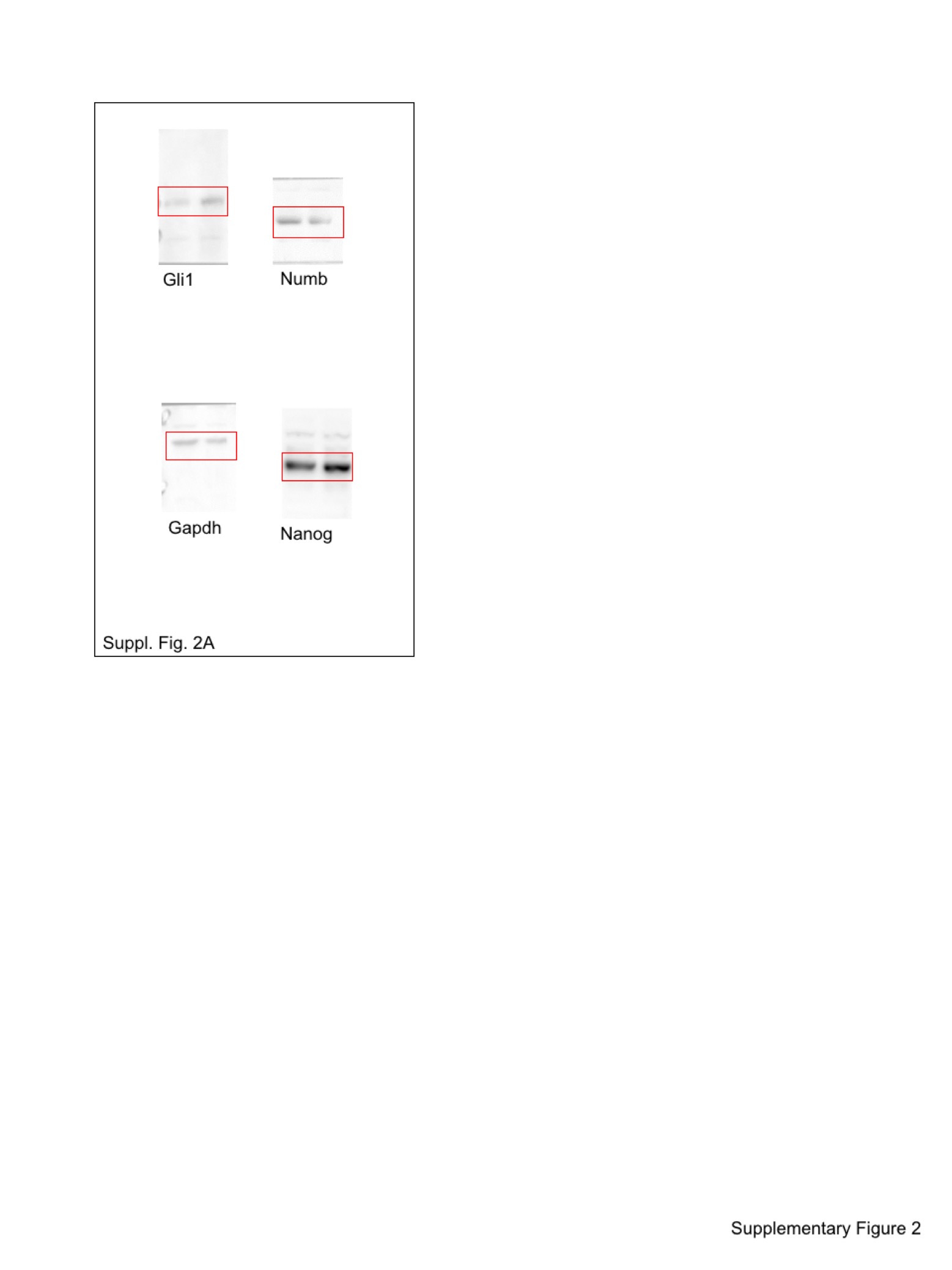

Supplement: Supplementary file 10 [file Image_10.JPEG]
